# Magnetic Confinement of a Bubble of Supercooled ^3^He-A

**DOI:** 10.1007/s10909-025-03328-w

**Published:** 2025-08-27

**Authors:** Luke Whitehead, Andrew Casey, Richard P. Haley, Petri J. Heikkinen, Lev V. Levitin, Adam J. Mayer, Xavier Rojas, Tineke Salmon, John Saunders, Alex Thomson, Dmitry E. Zmeev, Samuli Autti

**Affiliations:** 1 Department of Physics, Lancaster University, Lancaster, LA1 4YB UK; 2 Department of Physics, Royal Holloway, University of London, Egham, TW20 0EX UK

**Keywords:** Superfluid 3He, Magnetic confinement, A-B transition

## Abstract

We have designed and constructed a magnet surrounding a cylindrical volume of superfluid helium-3 to isolate a region of metastable, supercooled A phase, entirely surrounded by bulk A phase - isolating the ‘bubble’ from rough surfaces that can trigger the transition to the stable B phase. We outline the design of the experimental cell and magnet and show that the performance of the magnet is consistent with simulations, including the capability to produce the high field gradient required for generating a bubble. Future plans include the investigation of possible intrinsic mechanisms underpinning the A-B transition, with potential implications for early-universe cosmological phase transitions.

## Introduction

The process of nucleation of the B phase of superfluid helium-3 from the supercooled A phase is not well understood. Predictions made using homogeneous nucleation theory estimate a lifetime of the metastable state greater than the age of the observable universe [1], owing to the small free energy difference between the phases and large surface tension of the interface separating them [2]. In practice, this transition is observed to happen within hours [1].

The strong disagreement of the theory and experimental observations prompts investigation into the nucleation mechanisms that are responsible. It is well-established that external radiation sources [3] and surface roughness [4] can reliably trigger the transition, but there is potential for intrinsic mechanisms to contribute, with experiments taking place in the absence of (rough) walls and in conditions where ambient radiation is understood and minimised. The QUEST-DMC collaboration [5, 6] has already devised atomically smooth-walled nanofluidic cells to investigate this [7]. Here, we discuss a bulk approach: we have designed a magnet that allows creating an isolated region (a ‘bubble’) of supercooled A phase, completely surrounded by the stable A phase, eliminating interactions of the bubble with the container surfaces which are known to induce the phase transition. The size of the region, as well as the pressure and temperature of the volume, can be varied to investigate their effects on the nucleation rate.

The order parameter defining the phase of superfluid helium-3 is primarily dependent on pressure, temperature and external magnetic field. A spatially varying magnetic field in an experimental volume at constant temperature and pressure results in local phases. The magnet we have designed is such that a magnetic field minimum is generated within an elliptical volume with a magnitude that favours the B phase, surrounded by a region of higher field where the A phase is stable, and includes a compensation coil to eliminate interference with other magnetic fields produced above the experiment. Continuous monitoring of the temperature of the experimental volume will allow us to determine when the isolated elliptical volume - the bubble, has transitioned from the metastable A phase to the stable B phase due to the latent heat involved in the transition.

As the temperature approaches zero, superfluid helium-3 becomes absolutely void of impurities, making it an ideal candidate for studying nucleation theories of first-order phase transitions. The investigation of intrinsic mechanisms driving the A-B transition could have implications for early-universe cosmological phase transitions - important for future space-based interferometers that could detect the gravitational waves produced by such an event [8]. A new rapid intrinsic mechanism, for example, would act to reduce the expected gravitational wave intensity [8, 9].

## Magnet Setup

**Fig. 1:**
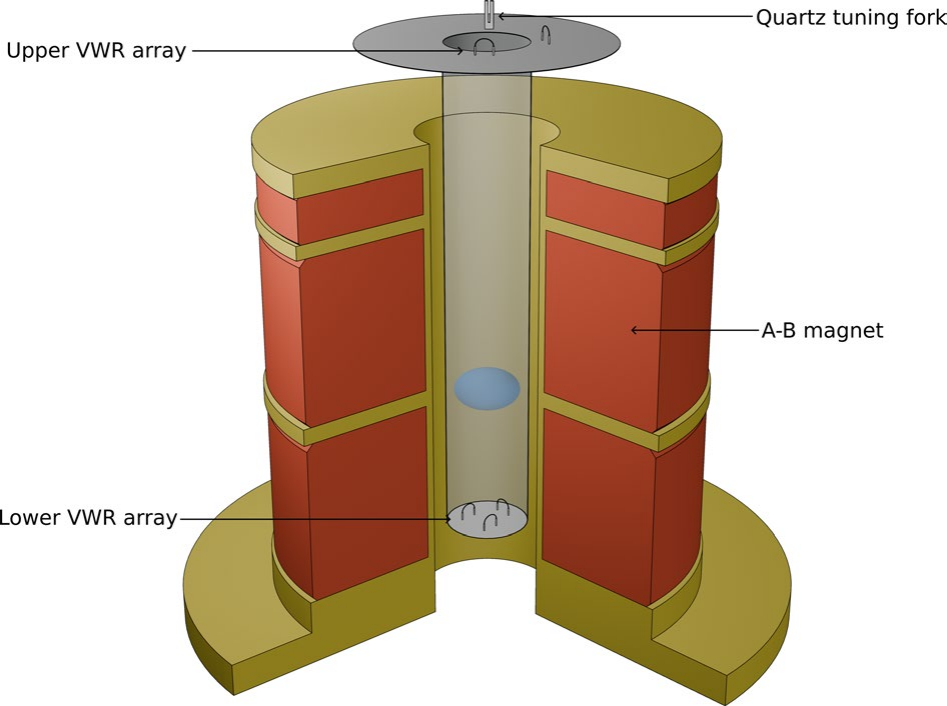
Schematic of the experimental cell and magnet. Above the main body of the inner cell but within the same volume are an array of VWRs and a quartz tuning fork for thermometry. The opposite end of the cell contains another series of VWRs for the same purpose. The A-B magnet used for generating the field gradient required to stabilise the bubble surrounds the helium-3 volume and is shown in red and gold. The approximate location and size of the metastable A phase bubble formed during the experiment is highlighted in blue and is surrounded by regular A phase. Not shown is the set of sintered-silver copper plates above the upper VWR array and tuning fork providing cooling for the sample, or the outer cell surrounding the experimental volume

The experimental volume (the ‘inner cell’) is a cylinder of Stycast 1266 epoxy reinforced paper, measuring 90 mm in length with an internal diameter of 14.9 mm, filled with ^3^He. The volume is in contact with silver-sintered copper plates which are cooled by nuclear demagnetisation, allowing the helium-3 to reach temperatures below 200 $\upmu$K. Surrounding this is another cylinder of helium-3 (the ‘outer cell’) acting as a thermal shield. Within the inner cell is a quartz tuning fork resonator [10] and an array of vibrating wire resonators (VWRs), used as heaters and thermometers [11] for the experiment, detailed in Fig. 1. The VWRs are small loops of superconducting wire, driven to oscillate at their resonant frequency by the Lorentz force when an AC current is supplied. The damping experienced by the wire as it moves through the superfluid can be determined from the Faraday voltage, measured by a lock-in amplifier in parallel with the drive current. As the upper VWR array (closest to the copper refrigerant) is in the B phase, the force measured by the wire is dependent on the quasiparticle density within the superfluid, allowing accurate measurement of the temperature within the experimental volume [12]. To heat the superfluid, a VWR is driven above the pair-breaking velocity, creating quasiparticles that travel ballistically through the bulk ^3^He [13].

The magnet used to stabilise a region of magnetic field within the superfluid consists of three coils; two main opposing solenoids to produce the required high field gradient (each 30 mm in height and 26 mm thick) and one smaller compensation coil (8 mm in height and also 26 mm thick). All are constructed from a single length of copper clad NbTi superconducting wire (Supercon 54S43) measuring 279 $\upmu$m in diameter, with each of the larger coils consisting of 8004 turns across 85 layers, and the smaller 2106 turns across 88 layers. The compensation coil ensures the field generated by the magnet does not interfere with the demagnetisation field required for cooling. The brass former for the magnet is attached to the inside of a radiation shield surrounding much of the dilution refrigeration unit and the demagnetisation stage of the cryostat, thermally anchoring the magnet to the still plate at approximately 0.5 K.

**Fig. 2:**
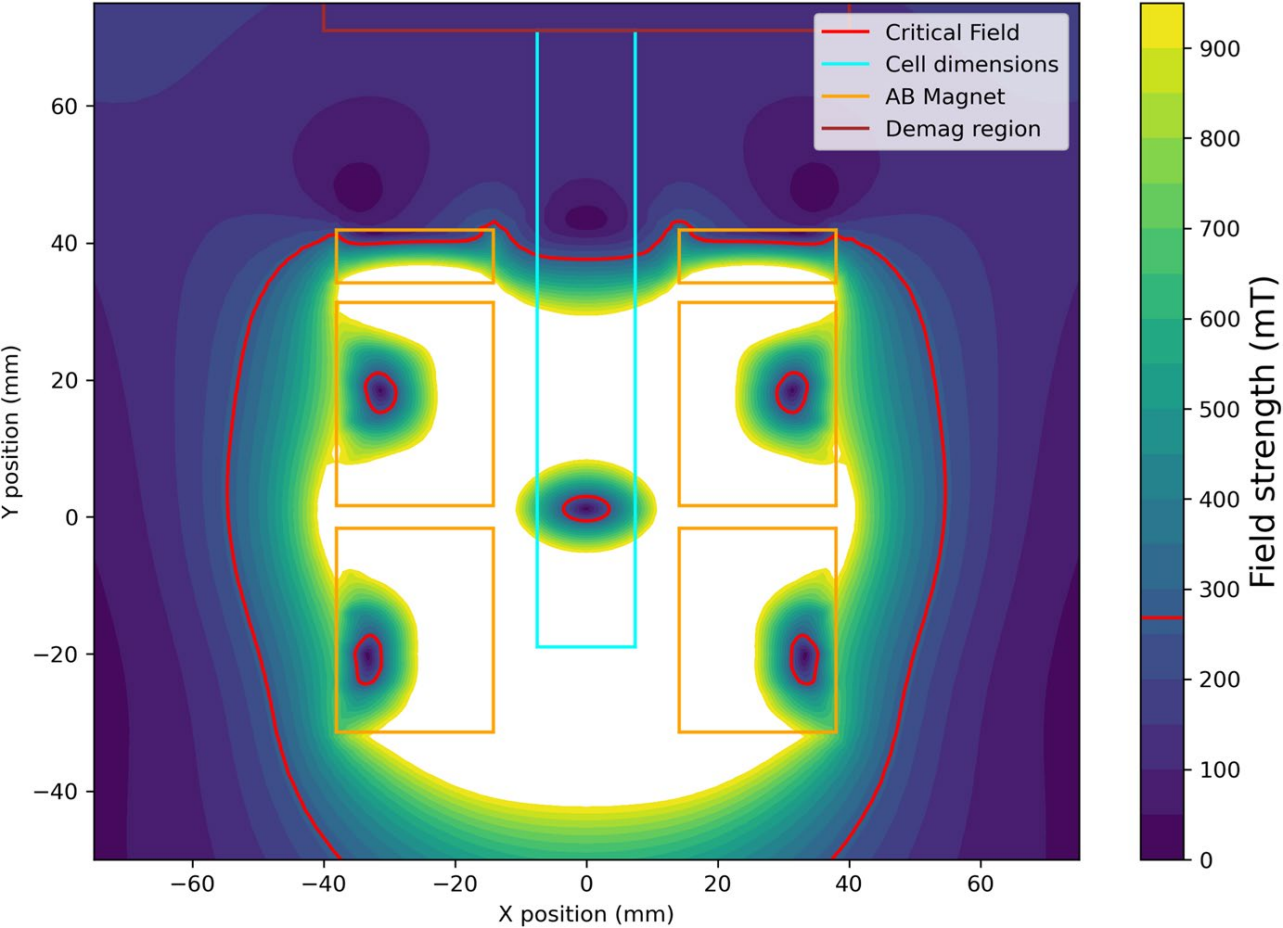
Simulated magnetic field profile. Outline of the experimental cell (blue) and magnet (orange) are shown. The critical field for creating an A-B boundary (red) is given for typical experimental conditions - in this case, a temperature of 0.75 $T/T_C$, a pressure of 20 bar and a 15 A current in the magnet, resulting in a region in the cell where B phase is preferred, surrounded by a volume of A phase. Regions where the field exceeds 900 mT are uncoloured

Figure 2 shows the simulated magnetic field profile in a typical experiment scenario. The critical field where the magnitude of the magnetic field favours the A phase under these conditions is highlighted in red.

**Fig. 3:**
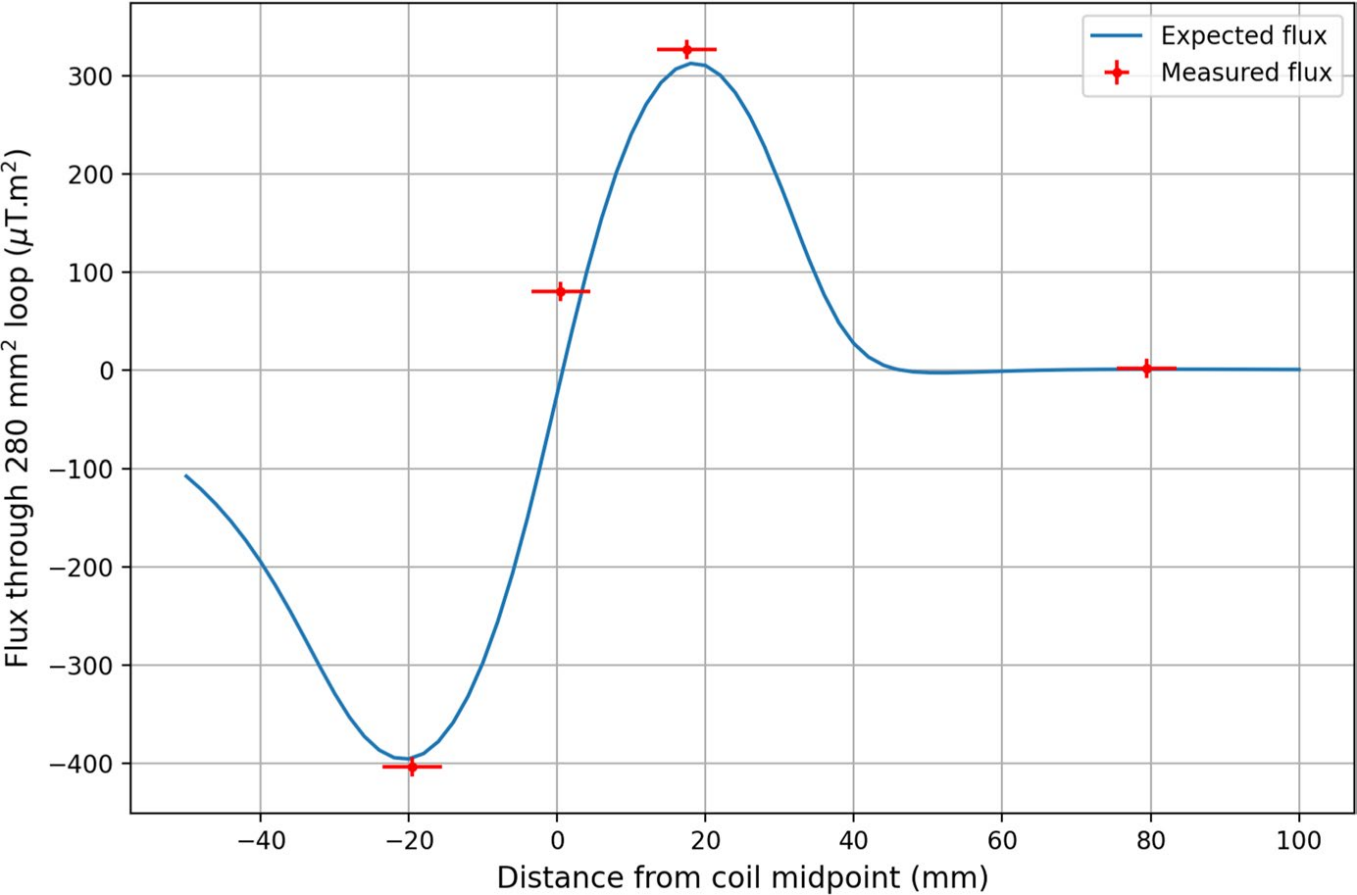
Predicted (blue) and measured (red) magnetic flux along the length of the magnet bore. Measured points from left to right correspond to the expected values for the field minimum, coil midpoint, field maximum, and far above the coil when a 10 A current is supplied to the magnet

A probe made up of a series of detection coils was developed to measure the magnetic field at four critical points: the expected maxima, the coil midpoint, and far away from the main volume, within the demagnetisation region. At all points, the measured field was within 5% of predictions (see Fig. 3), including complete compensation of the primary A-B solenoids in the demagnetisation region.

A probe made up of a series of detection coils was developed to measure the magnetic field at four critical points: the expected maxima, the coil midpoint, and far away from the main volume, within the demagnetisation region. At all points, the measured field was within 5% of predictions (see Fig. 3). Crucially, the primary A-B solenoids are completely compensated in the region above the main experimental volume, where nuclear demagnetisation of sintered copper plates is used to cool the helium-3 sample (for details on this process see Chapter 2.5 in [14]).

If we assume the radial component of the magnetic field produced by the magnet also has a $\pm 5 \%$ uncertainty, then the radius of the bubble will likewise be within 5% of the predicted value. A typical bubble with a radius of 5-6 mm can therefore be created to within 0.3 mm accuracy.

The magnet has been confirmed to operate above the required current threshold of 15 A for stabilising the B phase, reaching 19 A before quenching, and can run simultaneously with the main demagnetisation magnet without affecting its field.

## Discussion

We have developed a magnet capable of stabilising an isolated region of magnetic field favouring the B phase away from container walls. The initial testing shows the predicted magnetic field can be stabilised under planned experimental conditions. Previous measurements have shown how the presence of an A-B boundary provides a thermal resistance for quasiparticles [15–17]. By generating quasiparticles at one end of the cell using one resonator array and measuring the corresponding temperature rise at the other, we can determine the phase contained in the bubble, and therefore the lifetime of the metastable A phase. The latent heat released when the bubble undergoes the transition from the supercooled A phase to the B phase can also be used to monitor the life times [15]. This will allow us to investigate potential intrinsic nucleation mechanisms that trigger the transition from the supercooled A phase to the B phase by systematically varying the bubble size, temperature, and pressure and observing the time taken for the bubble to transition. We note that ambient radiation is a known process that can trigger the A-B transition. The QUEST-DMC collaboration has constructed a GEANT-4 model of several microkelvin refrigerators that can be easily applied to understanding the radiation conditions in this context as well [5, 6].

In the future, adding an extra gradient coil would allow moving the metastable bubble up and down inside the sample container. This would allow direct confirmation of the phase mechanically by moving the edge of the bubble to intersect with a mechanical probe. During the phase transition dynamics, mechanical measurements at the phase boundary could be used to extract the propagation speed and other out-of-equilibrium features of the process [17, 18] . These are particularly difficult to pin down theoretically, and would thus feed directly into related cosmological simulations of the early Universe. Finally, wide-band SQUID-based NMR techniques have been used by other groups to non-invasively determine the phase of the superfluid within a cavity [7]. Such a technique could be used to map the distribution of the order parameter within the metastable region. While the A-B interface has been studied extensively before, it has been in the presence of a third interface, in the form of a wall. Our bubble configuration opens up new opportunities for studying this exciting and ultimately pure 2D system.

## Data Availability

The data generated in this study have been deposited in the Lancaster University data repository at https://doi.org/10.17635/lancaster/researchdata/721
